# When a child dies: a systematic review of well-defined parent-focused bereavement interventions and their alignment with grief- and loss theories

**DOI:** 10.1186/s12904-020-0529-z

**Published:** 2020-03-12

**Authors:** Eline M. Kochen, Floor Jenken, Paul A. Boelen, Laura M. A. Deben, Jurrianne C. Fahner, Agnes van den Hoogen, Saskia C. C. M. Teunissen, Karin Geleijns, Marijke C. Kars

**Affiliations:** 1 Julius Center for Health Sciences and Primary Care, University Medical Center Utrecht, Universiteitsweg 100, 3584 CG Utrecht, The Netherlands; 2 Department Woman and Baby, Wilhelmina Childrens Hospital, Lundlaan 6, 3584 EA Utrecht, The Netherlands; 3 Department of clinical psychology, Utrecht University, Heidelberglaan 1, 3584 CS Utrecht, The Netherlands; 4 Arq Psychotrauma Expert Group, Nienoord 5, 1112 XE Diemen, The Netherlands

**Keywords:** Bereavement, Parents, Paediatrics, Systematic review, Models theoretical, Interventions

## Abstract

**Background:**

The availability of interventions for bereaved parents have increased. However, most are practice based. To enhance the implementation of bereavement care for parents, an overview of interventions which are replicable and evidence-based are needed. The aim of this review is to provide an overview of well-defined bereavement interventions, focused on the parents, and delivered by regular health care professionals. Also, we explore the alignment between the interventions identified and the concepts contained in theories on grief in order to determine their theoretical evidence base.

**Method:**

A systematic review was conducted using the methods PALETTE and PRISMA. The search was conducted in MEDLINE, Embase, and CINAHL. We included articles containing well-defined, replicable, paediatric bereavement interventions, focused on the parent, and performed by regular health care professionals. We excluded interventions on pathological grief, or interventions performed by healthcare professionals specialised in bereavement care. Quality appraisal was evaluated using the risk of bias, adapted risk of bias, or COREQ. In order to facilitate the evaluation of any theoretical foundation, a synthesis of ten theories about grief and loss was developed showing five key concepts: anticipatory grief, working models or plans, appraisal processes, coping, and continuing bonds.

**Results:**

Twenty-one articles were included, describing fifteen interventions. Five overarching components of intervention were identified covering the content of all interventions. These were: the acknowledgement of parenthood and the child’s life; establishing keepsakes; follow-up contact; education and information, and; remembrance activities. The studies reported mainly on how to conduct, and experiences with, the interventions, but not on their effectiveness. Since most interventions lacked empirical evidence, they were evaluated against the key theoretical concepts which showed that all the components of intervention had a theoretical base.

**Conclusions:**

In the absence of empirical evidence supporting the effectiveness of most interventions, their alignment with theoretical components shows support for most interventions on a conceptual level. Parents should be presented with a range of interventions, covered by a variety of theoretical components, and aimed at supporting different needs. Bereavement interventions should focus more on the continuous process of the transition parents experience in readjusting to a new reality.

**Trial registration:**

This systematic review was registered in Prospero (registration number: CRD42019119241).

## Background

After the death of an infant, or child, parents are left with an intense and overwhelming sense of grief [1–3]. Parents experience an accumulation of feelings of loss from the child’s initial diagnosis, through the progressive deterioration in the child’s condition, and eventually, to the death of the child [4]. In addition to their own feelings of grief, parents also experience the burden of grief from the dying child and their siblings [3]. Grief is a normal reaction to the loss of a child. For most parents, moderate support from regular health care professionals (HCPs), and relatives, is sufficient in helping to cope with feelings of grief [5]. However, around 10 to 25% of parents experience a serious disruption in emotional stability, which may result in poor psychosocial outcomes and adverse mental and physical health effects [6, 7].

A growing body of literature demonstrates that HCPs recognise parents’ need for support in handling feelings of loss and grief [8–10]. This has resulted in an increasing number of interventions in practice aimed at all bereaved parents and provided by regular HCPs [10]. Although care standards state that providing bereavement care to parents is an important aspect of end-of-life care, such care is not yet routinely implemented in most hospitals [7, 11]. This might be due to the fact that HCPs often feel ill equipped to provide bereavement care [12]. Another explanation might be that bereavement interventions based in practice do not contain clear guidelines or protocols, making them difficult to standardise [13]. The assumption is that clear protocols and guidelines make interventions replicable for other HCPs. An overview of, clear, replicable interventions, containing guidelines and instructions, could lead to improved implementation and appropriate care delivery to all bereaved parents. This is because the availability of evidence-based practice guidelines could enable HCPs to feel more equipped [12]. However, such an overview is currently missing.

Another characteristic of this practice-based nature of the interventions is that theoretical and empirical support are often unclear or not provided at all [10, 14, 15]. Theoretical understanding is an essential ingredient in developing, evaluating, and implementing behavioural interventions and best clinical practices [16]. A social theory can be seen as a set of statements that explain aspects of social life, and which demonstrate how people conduct and find meaning in daily life [17]. However, the theoretical field of loss and grief is still evolving. Nevertheless, several theories have been put forward to provide a supporting structure to the theoretical understanding of the process of grief [18–30]. Understanding how different elements of interventions might relate to, or rely on, such theories, could improve our understanding of the underlying mechanisms of these interventions and provide an indication of their effectiveness.

This review will provide an overview of well-defined bereavement interventions performed by regular HCPs, and aimed at supporting parents in coping with loss, during both the end of their child’s life and after their child’s death. Furthermore, we will provide an overview of their effectiveness and whether the bereavement interventions currently practiced are substantiated by theory about loss and grief, and, as such, provide a theoretical basis for the effective elements of bereavement interventions.

## Methods

### Design

The field of paediatric palliative care is relatively young and so clear terminology is yet to be established. Therefore, we used an iterative method for constructing a search strategy: Palliative cAre Literature rEview iTeraTive mEthod (PALETTE) [31]. In addition, our method complied with the Preferred Reporting Items for Systematic Reviews and Meta-Analyses (PRISMA) [32]. This systematic review was registered in Prospero (registration number: CRD42019119241).

### Databases and searches

The first articles were identified through a preliminary search in PubMed and via expert advice from senior researchers in the field of paediatric palliative care and bereavement. From these articles, different synonyms were gathered and terminology became clearer, a process known as ‘pearl growing’. As a result, articles were identified which were referred to as golden bullets because they met all inclusion criteria and thus should be included in the review. These processes resulted in additional searches. The process of pearl growing, identifying such new articles and adjusting the search string conducted in collaboration with an information specialist, was repeated until the search was validated [31]. That is, when all golden bullets were identified in the results of the search. Subsequently the information specialist involved conducted the final structured literature search in the following databases: MEDLINE, Embase, and CINAHL. See Additional file 1 for the full search strings.

### Study selection

The studies that were published in peer reviewed English language journals between January 1, 1998 and November 15, 2018, were included when they contained a well-defined bereavement intervention, offered by regular HCPs, to parents of deceased children or children with a life limiting condition at the end-of-life phase. This period of time was chosen because palliative care was formalised in a definition by the World Health Organization (WHO) in 1998, providing a consensus around the term ‘palliative care’. Interventions were defined as an intentional act performed for, with, or on behalf of, a parent or parents. An intervention must consist of well-defined, concrete proceedings. This means it can be replicated by other HCPs and is supported by instructions, a manual, training, a program or other supporting documents. We defined regular HCPs as professionals working in neonatal, or paediatric, care, where in their daily tasks, they are confronted with palliative care and care for loss and bereavement, without having necessarily received specialist training in these domains. Furthermore, interventions aimed at complex grief were excluded, since most parents do not require specialised services and such interventions are mostly performed by specialists on bereavement care. Full inclusion, and exclusion, criteria are listed in Table 1. When the full text was not available online, or when it was unclear whether the practices described were supported by a protocol or supporting documents, the first author of the article was contacted by email and requested to send additional information or a copy of the article. Both the title and abstract, and full text screenings, were performed by two researchers independently (EK, FJ), supported by the web-based screening program Rayyan (https://rayyan.qcri.org/welcome). Disagreements were resolved in dialogue with the research team. All the articles included were reference checked for additional relevant studies.

**Table 1:** Inclusion and exclusion criteria

Inclusion criteria:	
• Articles containing well-defined bereavement interventions offered by regular HCPs to parents of children who have died or those children in the phase of receiving palliative care.	
• Interventions aimed at consoling intense feelings of grief during the end-of-life phase or after the loss of a child. Bereavement care may also occur before the death of the child, for example from the moment the condition of the child is deteriorating and death is imminent.	
• Studies must address interventions defined as: Intentional acts performed for, with, or on behalf of, a parent or parents. An intervention must consist of well-defined, concrete proceedings. This means it can be replicated by other HCPs and is supported by instructions, a manual, training, a program or other supporting documents. Our definition is based upon the definition of interventions used by the World Health Organization [33].	
• Studies must address regular HCPs defined as: All types of health care professionals who primarily provide care and/or treatment and, therefore, do not specialise in bereavement care.	
• Research in the field of paediatrics and neonatology.	
• Articles published in a peer reviewed journal.	
• Studies published in English.	
Exclusion criteria:	
• Review articles.	
• Articles published before 1998.	
• Articles containing interventions that focus on complex grief and complex bereavement care.	
• Articles which solely include prenatal death and stillbirth, defined as: No signs of life at or after 28 weeks’ gestation. No occurrence of circulation outside of the uterus.	

### Data extraction and quality assessment

Data on baseline characteristics, participants, interventions, and outcomes were extracted by three researchers (EK, KG, FJ) using a predesigned form based on Schulz’s intervention taxonomy [34].

The quality assessment was performed by two researchers independently. The trials were assessed using the Cochrane risk of bias tool (KG, AvdH) [35], observational studies with an adapted risk of bias tool based on the Cochrane risk of bias assessment tool (KG, AvdH) [36], and qualitative studies were assessed with the COnsolidated criteria for REporting Qualitative research (COREQ) (FJ, EK) [37], recommended by Cochrane Netherlands. The total scores ranged from 0 to 7 in the trials and observational studies, and from 0 to 32 in the qualitative studies. The quality appraisals did not affect inclusion in the review due to the explorative nature of this systematic review, and also due to the fact that articles containing low appraisal scores could still contain valuable interventions and thus be relevant for the study aim [38].

### Synthesis of grief theories

The interventions were compared with a theoretical synthesis, in order to compensate for the expected lack of evidence for most interventions, and to evaluate the possible effectiveness. Since there is not a singular dominant theory on grief [16], leading theoretical models have been identified using a pragmatic approach. At first, experts in the field of bereavement (PB, MK, EK) and palliative care (MK) were consulted, preliminary searches were conducted in Google scholar and Medline, and; a compendium on bereavement was consulted [39]. Secondly, a pragmatic search was conducted in Medline using keywords such as grief, loss, bereavement, theory and equivalents (EK). Thirdly, the theories identified were validated by experts (PB, MK). They aimed for articles that showed the variation in bereavement theories and were a reflection of the most accepted theories from several different domains [18–30]. By doing so an overview of the leading theoretical concepts available was developed, which were extracted from the theoretical articles, clustered into communal theoretical concepts, and labelled accordingly. Most theories on grief emphasise that bereaved families need to adjust from the ‘old world’ to the ‘new reality’ [18–21, 23, 26–30], where the deceased is no longer physically present. This readjustment can be seen as a continuous process that takes months to years to complete, while the grief, itself, may never be resolved. The theories propose different approaches to how this adjustment is achieved. However, when comparing the leading theories we found that most theories have several key concepts at their core. This offered the opportunity to synthesize the theories on a conceptual level and, as such, capture the core mechanisms of most theories. These core mechanisms create the ‘how’ in which the theories explain the process of readjustment to the new reality. The synthesis of theories resulted in five concepts: anticipatory grief; an attachment to working models and plans; appraisal processes; coping behaviours, and; continuing bonds. These five concepts will be discussed in the following section. Importantly, these concepts do not represent elements of a sequential process, but rather elements of adjustment that may be re-addressed over time. The Additional file 2 displays how the theoretical concepts are formed, based on different theoretical articles.

Anticipatory grief refers to feelings of loss and grief before an imminent loss [30]. It involves forms of coping and reorganisation prior to loss and death, managing conflicting demands, facilitating a ‘good’ death, and preparedness. Preparedness comprises several different dimensions such as medical, psychosocial, spiritual, and practical dimensions [25]. Preparedness may help informal caregivers in coping with grief at a later stage.

Concepts concerning attachment working models and plans enhance multiple types of plans, namely: internal plans such as personal plans which may help a person understand their environment [27, 28]; relational plans such as how the self relates to others [26, 28, 30], and; attachment plans such as those created in early childhood and which guide a person in forming attachment bonds with others [19, 23]. Such plans make the world understandable, recognisable, and predictable. However, sometimes they do not match reality, for example when a child dies. This causes a severe stress reaction. This new reality must be incorporated into the existing plans to establish a new stable situation [18, 20].

Appraisal systems are set up when a new situation needs to be evaluated. In the situation of the loss of a child, the appraisal systems conclude the fact that the reality does not match the existing plans [19, 20, 23, 24, 26]. Appraisal systems will then be active until new plans are developed [26], or the old plans are revised [26, 30]. The loss is then incorporated into the autobiographical memory and a revision of self-identity can take place [18, 27].

Stressful situations are managed by employing helpful coping behaviours [18, 20]. Different coping styles exist, such as those focusing on the problem or the emotion [24]. Some coping styles may be orientated towards loss or restoration [21, 30], while some strategies may seek to make meaning out of the experience [28]. The reaction and coping behaviours differ between individuals and depend upon several factors including context and personality [26]. Effective coping includes the ability to shift, flexibly, between different coping strategies [20, 21, 27].

Finally, the concept of continuing bonds refers to an ongoing relationship between the individual and the deceased [21, 22, 26].

## Results

**Fig. 1:**
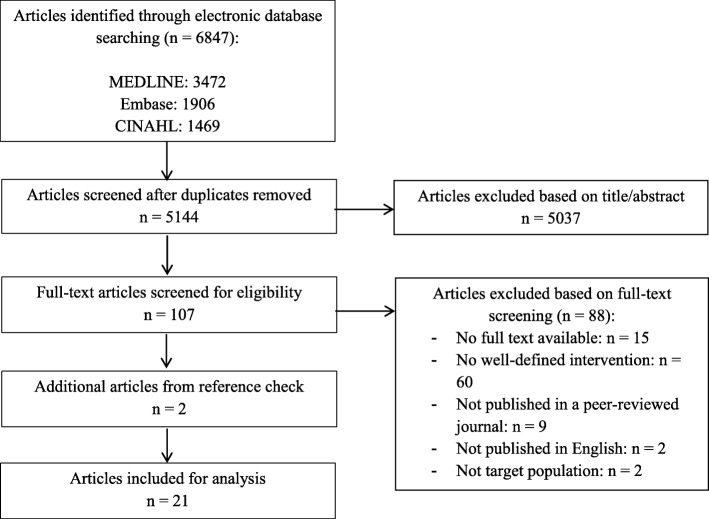
Study flow

The search yielded 5144 unique articles, of which nineteen met the inclusion criteria [40–58] and two were added following an additional reference check (Fig. 1) [59, 60]. Twelve articles represented empirical data drawn from the interventions of bereavement care programmes. Of these, four represented quantitative studies [40–43], six represented qualitative studies [44–49], and two represented studies which included both quantitative and qualitative outcomes [50, 51]. Nine articles were descriptive in nature [52–60]. These articles contained well-defined bereavement interventions, yet the interventions were not tested empirically and, therefore, the outcomes could not be provided. An overview of all the articles included is provided in Table 2. Quality appraisals ranged between 2 and 5 for trials and observational studies, and between 8 and 21 for qualitative studies. Quality scores on all studies can be found in Table 2. Qualitative studies received higher appraisal scores.

**Table 2:** Baseline characteristics

**Quantitative studies**							
**Author/year/country**	**Study type**	**Aim of the study**	**Setting**	**Sample**	**Method of data collection**	**Outcomes measures**	**Quality**
Aho et al. (2011) [40], Finland	RCT Follow-up program vs. usual care	To evaluate a bereavement follow-up intervention for fathers, by comparing grief reactions and to explore their experiences with the program	Intensive care unit, maternity ward, and emergencyroom in five university medical centres	Fathers of children who died at age ≤ 3 years	1. Hogan Grief Reactions Checklist 2. Questionnaire measuring social support provided by HCPs and peer supporters 3. Questionnaire measuring fathers experience with the follow-up program	1. Despair, panic behaviour, personal growth, blame and anger, detachment, disorganisation. 2. Affect, affirmation, aid from HCPs and peer supporters 3. The implementation of the program	2 out of 7
Meert et al. (2014) [41], USA	Observational study	To evaluate the feasibility and perceived benefits of conducting physician-parent follow-up meetings	Seven children’s hospitals, oncology units	Critical care physicians, bereaved parents of children who have died in the PICU, relevant others	Survey (items on Likert scale and open-ended questions)	1. Physician adherence to the framework 2. Experiences with follow-up meeting	4 out of 7
Nikkola et al. (2013) [42], Finland	Observational study	To describe mothers’ experiences with the bereavement follow-up program	Intensive care unit, maternity ward, and emergencyroom in five university medical centres	Mothers of children who died at age ≤ 3 years	1. Questionnaire measuring social support provided by HCPs and peer supporters 2. Questionnaire measuring mothers experience with the follow-up program	1. Affect, affirmation, aid. 2. The implementation of the program	5 out of 7
Raitio et al. (2015) [43], Finland	RCT Follow-up program vs. usual care	To explore the effects of a bereavement follow-up intervention on mothers’ grief	Five university medical centres	Mothers of children who died at age ≤ 3 years	1. Hogan Grief Reactions Checklist	1. Despair, panic behaviour, personal growth, blame and anger, detachment, disorganisation.	2 out of 7
**Qualitative studies**							
**Author/year/country**	**Study type**	**Aim of the study**	**Setting**	**Sample**	**Method of data collection**	**Outcomes**	**Quality**
Aho et al. (2011) [44], Finland	Generic qualitative study	To evaluate the experiences and suggestions for further improvement of a bereavement follow-up program intervention	Perinatal and neonatal units	HCPs who were appointed to care for a child who died at age ≤ 3 years	Open-ended questionnaire and individual telephone interviews	1. Experiences with the bereavement follow-up program 2. Ideas to improve the bereavement follow-up program	14,5 out of 32
Berrett-Abebe et al. (2017) [45], USA	Generic qualitative study	To understand parents’ experiences with participation in a hospital-based bereavement support program following the loss of a child to cancer	Tertiary care centre, Department of paediatric haematology/oncology	Parents of children who have died of cancer	Focus group	1. Experiences with medical team during child’s illness 2. Experiences with bereavement follow-up program after child’s death 3. Experiences of other bereavement support	19,5 out of 32
Brink et al. (2016) [46], Denmark	Generic qualitative study	To explore parents’ experience of a follow-up meeting	University hospital, Paediatric Intensive Care Unit	Parents of children (aged 0–16) who have died in the PICU	Individual face-to-face interview	1. Experiences around a follow-up meeting	19 out of 32
Darbyshire et al. (2012) [47], Australia	Generic qualitative study	To explore the experiences of parents who participated in a nurse-led telephone follow-up support program in paediatric oncology.	Regional women’s & children’s hospital, paediatric oncology unit	Parents of children who have died from an oncology-related condition	Individual face-to-face interview	1. Experiences with a follow-up support program	21 out of 32
Eggly et al. (2011) [48], USA	Generic qualitative study	To describe a framework to assist PICU physicians in conducting follow-up meetings	Seven children’s hospitals, oncology units	Critical care physicians and bereaved parents whose children died in the PICU	Individual interviews by telephone	1. Experiences with follow-up meetings	8 out of 32
Meert et al. (2011) [49], USA	Generic qualitative study	To investigate physicians’ experiences and perspectives regarding follow-up meetings	Seven children’s hospitals, oncology units.	Critical care physicians	Individual interviews by telephone	1. Experiences with follow-up meetings 2. Ideas for future follow-up meetings	17 out of 32
**Mixed method study**							
**Author/year/country**	**Study type**	**Aim of the study**	**Setting**	**Sample**	**Method of data collection**	**Outcomes**	**Quality**
Michelson et al. (2013) [50], USA	Mixed method study	To describe implementation of, reflections on, and address barriers for a PICU bereavement photography program, according to HCPs	Children’s hospital, PICU	HCPs who cared for children at PICU who met one of following criteria: impending death, planned withdrawal of life-sustaining therapies with an expectation of a sudden death, examination consistent with brain death	Questionnaires (closed and open-ended questions)	1. Experiences with a bereavement photography program 2. Ideas to improve the program	4 out of 7 15,5 out of 32
Oliver et al. (2001) [51], USA	Mixed method study	To explore experiences with a bereavement support program	Regional children’s hospital, paediatric trauma centre	Families of children who have died in the paediatric trauma centre and parental supporters	Survey and individual interview	1. Experiences with a bereavement support program	4 out of 7 10 out of 32
**Descriptive articles**							
**Author/year/country**	**Study type**	**Aim of the study**	**Setting**	**Target population**	**Method of data collection**	**Outcomes**	**Quality**
Aho et al. (2010) [52], Finland	Descriptive article	To describe the development and implementation of a bereavement follow-up intervention for grieving fathers	Five university medical centres, perinatal and neonatal unit	Fathers of children who died at age 3 or younger	N.A.	N.A.	N.A.
Cook et al. (2002) [53], UK	Descriptive article	To review local bereavements support practices over the last 5 years	Regional hospital, PICU	Parents of children who have died unexpectedly in the PICU	N.A.	N.A.	N.A.
Edi-Osagie et al. (2005) [59], UK	Descriptive article	To describe a template for a bereavement service	Tertiary care centre, NICU	Parents of children who have died in the NICU	N.A.	N.A.	N.A.
Gibson et al. (2011) [60], USA	Descriptive article	To describe the development of a NICU bereavement program	University hospital, NICU	Parents of children who have died in the NICU	N.A.	N.A.	N.A.
Levick et al. (2017) [54], USA	Descriptive article	To summarize and evaluate a comprehensive approach of bereavement services to NICU families and education/support to NICU staff	Regional children’s hospital, NICU	Parents of children who have died in the NICU	N.A.	N.A.	N.A.
Morris et al. (2016) [55], USA	Descriptive article	To discuss the goals of bereavement care and the need to standardize bereavement care in the paediatric setting, and describe their hospital wide bereavement model	Hospital, paediatric setting	Families of children who have died in a paediatric setting	N.A.	N.A.	N.A.
Reilly-Smorawski et al. (2010) [56], USA	Descriptive article	To evaluate experiences of both individuals and couples with a bereavement support group	Tertiary Centre, NICU	Parents of new-borns or infants who have died in the NICU.	N.A.	N.A.	N.A.
Snaman et al. (2017) [57], USA	Descriptive article	To review the three primary pillars of a parent-inspired and parent-derived bereavement program	University children’s hospital, oncology and palliative care unit	Parents of children who have died of cancer	N.A.	N.A.	N.A.
Stastny et al. (2016) [58], USA	Descriptive article	To provide a practical guideline for public health nurses (PHN) in performing home visits to bereaved parents	Home visits	Parents of children who have died of sudden infant death syndrome	N.A.	N.A.	N.A.

The twenty-one articles included fifteen unique bereavement interventions, identified with the letters of the alphabet A through to O. Two interventions were described in multiple articles (A and G). The intervention characteristics are summarised in Table 3.

**Table 3:** Intervention characteristics

**Author/year/ country**	**Interventionist**	**Intervention**		**Outcomes**	**Development**	**Implementation**	**Theoretical support**
		**Outline**	**Components**				
A. Aho et al. (2010) [52], Aho et al. (2011a) [40], Aho et al. (2011b) [44], Nikkola et al. (2013) [42], Raitio et al. (2015) [43], Finland	Peer supporters and HCPs	- Support package at discharge after the child’s death - Peer support one week after the child’s death - Follow-up contact by HCP after 2–6 weeks	- Support package - Peer contact - HCP contact	Outcomes fathers (Aho et al. 2011a): - Experienced most affect and emotional support, more from peer supporters than from HCPs. - Most fathers responded that the follow up contact helped them in coping, timing of contact was appreciated - Lower values in all dimensions of grief except for personal growth in the intervention group Outcomes mothers: - No significant differences in grief reactions between intervention - and control group (Raitio et al. 2015) - Mothers received most affect, moderate affirmation, and little aid from HCPs and peer supporters (Nikkola et al. 2013) - Follow-up contact helped mothers in coping (Nikkola et al. 2013). Outcomes HCPs (Aho et al. 2011b) - Follow-up contact important element of care, but also stressful and difficult - Implementation possible due to positive attitude HCPs, resources inadequate - Intervention increased cooperation between HCPs and peer supporters	- Baseline study on current bereavement support systems - Systematic review - Expert panel - Clinical experience and patient perspective	- Training for implementers - Training to use the intervention was provided for peer supporters and HCP	Not mentioned
B. Berrett-Abebe et al. (2017) [45], USA	Social worker (program coordinator) and clinician	Two years bereavement program: - Comfort basket 3–4 weeks after death - Phone call/note: 2 weeks, 1 month and 2 months after child’s death, annually on birthday and anniversary - Letters: at 3,6,10,12,18,24 months after death	- Comfort basket - Phone calls - Letters - Info sheets - (Anniversary-)cards	Identified themes: (1) Lived experience of grief; grief is intense, long-lasting, varies day by day, different for everyone. Relationships could become strained or a comforting source of support. (2) Relationships HCPs: Being treated like family, human connectedness and compassion (3) Hospital-based bereavement support: Feeling of not being forgotten by HCPs, parents appreciated talking to HCPs who were not afraid of talking about their traumatic experiences. Parents valued the content of the letter, comfort basket and materials. (4) Preferences extended bereavement care: ongoing, flexible, annual informal gathering, formalizes peers support contact	- Development by multidisciplinary working group in oncology - Based on social support theory, input from parents, and clinical knowledge	Not mentioned	Stress and coping social support theory: social support helps individuals manage stressful situations by improving coping responses
C. Brink et al. (2016) [46], Denmark	PICU physician and nurses	90-min follow-up meeting at the PICU, 4–8 weeks after the loss of the child: 45 min to discuss medical topics (physician and nurse) and 45 min to discuss care and dealing with everyday life (nurse)	- Follow-up meeting	Identified themes: (1) Turning back: stressful and unpleasant to return to PICU, no prior expectations, valuable to see HCPs affected by the child’s admission. (2) Framework meeting: ambience calm and oppressive or good and emotional (with focus on parents). Participation of nurses was valued, parents experienced more tenderness when the physician left. (3) Relations HCP: relationship with staff makes return to PICU good experience, eg. being recognized and mentioned by name. (4) Closure: meeting was experienced as closure of the course in the PICU	Not mentioned	Not mentioned	Not mentioned
D. Cook et al. (2002) [53], UK	Trained counsellor and doctor	- Information letters for parents - Encouragement of families to seek support - Follow-up meetings 8–12 weeks after child’s death	- Follow-up meeting - Personalized information - Encouragement	Not applicable	Trained counsellor is available	Not mentioned	Not mentioned
E. Darbyshire et al. (2012) [47], Australia	Designated nurse	- Information folders, containing contacts, readings and practical advice - Attending the child’s funeral - Phone calls until 13 months after death - Cards at special times such as birthday.	- Information folders - Attending the funeral - Follow-up phones calls - Sending cards	- All parents received the follow-up calls and were satisfied with the length of the follow-up program. - All parents were positive about the telephone follow-up program and valued the opportunity to share memories with someone who knew their child - Personalized cards and letters felt as an acknowledged of the important relationship with the hospital.	The intervention was based on a literature search and a focus group.	- Bereavement education and training twice a year. - Bereavement case file is created, including a photo, call plan, and copies of correspondence.	Not mentioned
F. Edi-Osagie et al. (2005) [59], UK	Bereavement Care Team (BCT): team member that had most contact with the parents before death, or the one with the lightest case load.	*Prior to death:* Introducing member of BCT, counsellor, and chaplain. Offer blessing or religious ceremony and access to bereavement suite. *Immediate period following death:* Literature/information is provided, clothing from bereavement room nursing the baby, provide cold cot. Help planning the funeral and attend, card is sent. Provision of memory box and keepsake bag. Helps explaining the death to the siblings. 24 h telephone support available. Advice on financial matters and social benefits. *Follow up:* Home visit to all bereaved parents, flowers are sent after 6 weeks. Annual remembrance service.	- Blessing/religious ceremony - Bereavement suite - Memory book - Pictures - Keepsakes - Information letters - Provide cold cot - Help in arranging the funeral - Memory box and keepsake bag - Access to telephone support 24/7 - Financial advice and benefits - Follow-up visit - Flowers sent - Annual remembrance service	Not applicable	Members of bereavement care team have undertaken counselling courses, educational workshops, and workshop on how to train others.	Regular education sessions for HCPs, written guidance.	Not mentioned
G. Eggly et al. (2011) [48], Meert et al. (2011) [49], Meert et al. (2014) [41]	Physicians of the PICU who are trained in conducting follow-up meetings.	Framework follow-up meeting: - Invitation at discharge and after one month - Card/call after one month, evaluating preferences for meeting and planning - Follow-up meeting (1 h) - After meeting: thank you note, supportive information - Debriefing for HCPs	- Follow-up meeting - Supportive materials and information - Phone call and card	- Physicians’ participation in follow-up meetings: never (33%), 1–5 meetings (31%), > 5 meetings (36%). Attendants participated more often than fellows. - Parents perceived the meeting as helpful for themselves (92%), for others (89%) and in coping with the future (78%) - Physicians stated that they adhere to the framework (75%), consider the framework easy to use (92%), beneficial for parents (92%) and for themselves (89%)	Eggly S (2011): Framework is based on the experience and perspectives of bereaved parents and paediatric intensive care unit physicians.	Physician participants were trained to use the follow-up meeting framework via face-to-face or web-based small group sessions. Training included: education on bereavement processes and the framework, simulated follow-up meetings and interactive discussions	Not mentioned
H. Gibson et al. (2011) [60], USA	Staff of the NICU (mostly nurses and social workers), all bereavement council members.	*Prior to death:* professional photography, offer baptism, discuss end of life preferences *After death of the child*: Give teddy bear; inform about memory box and follow-up contact; provide folders and reading material. *Follow-up contact:* 6 fixed times, from 1 day through 1 year. Card schedule: 6 cards on special days. Twice a year a memorial service. Parents are invited the first two years after death.	- Washing/holding the child - Baptism/religious ceremonyavailability of family room - Hand−/footprints and lock of hair - Memory box (includes CD with photos, bracelets, rings, shell from baptism, any bedside belongings) - Follow-up cards (including butterfly ornament) and calls - Family support folder - Casket - Remembrance ceremony	Not applicable	Practice-based and on the personal experiences of one nurse. Several nurses and 2 social workers attended the Resolve Through Sharing (RTS) training by Bereavement Services	- Checklist in medical file - Education new employees and one-a-year education fair - Monthly council meeting	Not mentioned
I. Levick et al.(2017) [54], USA	Neonatologist and designated staff member (primarily nurse) with support from BCT	*When neonate just died:* inviting loved ones, hold and bathe child, preserving infant’s bedside till parents are ready to remove it. Keepsakes even if parents are uncertain. In that case, hospital stores the keepsakes. The ability to let parent help with making keepsakes. Checklist of services that can be provided. *Follow-up program*: call schedule; within days, at 2-3 weeks, after three weeks adjusted to wishes parents until 12 months after death. Card schedule: standard within 2 weeks and at 11 months. Other moments adjusted to wishes of parents.	- Hold/bath child - Sympathy cards - Follow-up phone calls - Photos of the child - Hand−/foot−/head prints of the child, could be combined with hand of the parent/sibling - Sibling support program - Bereavement information folder - Certificate of life - Beaded name bracelet - Memory stone - Locket of hair - Seashell used for baptism - Bereavement gown and/or gown crafted from donated wedding dresses - Escort parents/siblings to the car - Keepsake box for siblings (storybooks, stuffed animals, memory stone, hand−/footprints	Not applicable	Literature review	- The intervention is coordinated by the NICU Bereavement Care Team (BCT). - Bereavement/keepsake checklist is used by all personnel. - BCT Nurse reports personal information and dates, and designated nurse appointed in spreadsheet.	Not mentioned
J. Michelson et al. (2013) [50], USA	Photographer who has specific expertise in bereavement photography and training in bereavement support.	Photographer is updated on medical/ family situation of family by HCP. Taking photographs of patient and family without posing.Preparing album in documentary style and deliver album to family.	- Photograph album of patient and family in documentary style	- HCPs thought parents were grateful for photos (85%), and photos made HCPs feel better about their role (70%) and did not take too much time (85%). - Positive: impact program on families and HCPs - Barriers: funding, availability photographers, informed consent parents	Program was based on a bereavement photography program in NICU and adjusted with input from multidisciplinary group.	Education of staff members about the program through presentations at regular meetings, information provided online and individually.	Not mentioned
K. Morris et al. (2016) [55], USA	Program coordinator, social workers and a nurse practitioner	- Newly bereaved families are mailed a bereavement packet (includes a formal condolence letter, a psycho-educational bereavement guide, a flyer outlining upcoming seminars at the hospital, and a list of online programs). - Seminars for parents about coping with grief and 8-week support group each spring. - Availability of support groups, individual counselling, telephone support, and memorial service	- Condolence letter - Memorial events - Educational guide (booklet and on website) - Seminars about coping with grief - Support group - Workshop for parents and siblings - Telephone support - Referral and resource information	Not applicable	The program is developed by parents and staff. The bereavement program was modelled on the bereavement program developed at a near cancer institute where education, guidance and support were identified as the primary constructs.	Quarterly seminars for staff, offered by the bereavement Task Force, about grief, bereaved families, and self-care for clinicians .	The psycho-educational bereavement guide “When Grief is New”, is based on cognitive behaviour theory principles.
L. Oliver et al. (2001) [51], USA	Chaplain	- First meeting at hospital just after child’s death (religious rituals are offered, parents are provided with informational brochures) - Second meeting at funeral or the families’ home after one month - Third meeting: educational dinner with the family and 15 supporters (eg friends/family), within two months after death	- Information brochure - Information video for surviving children - Attending funeral - Home visit - Educational event with supporters	Parent Survey: - Time in hospital: staff were reported sensitive to the child and parents (90% & 93%), prepared parents for death (81%), and the treatment was understandable (90%). - Chaplain’s first visit: parents wanted a meeting, the meeting was helpful, and answered questions (80, 90, 78%). - Meeting with supports: Supporters remembered the child (91%), accepted adjustment time (89%), and called, visited, take out and wrote more (73%) - Supporters survey: The meeting helped supporters understand parents’ journey (95%), prepared to care (82%), made it likely to use advise (82%), supporters took specific actions to remember the child (69%), accepted adjustment time (94%), and called, visited, took out, wrote more (78%). Observations on the support network: 63% took actions to remember the child, 50% accepted adjustment time, 31% called, visited, took out and wrote often, and 77% reported ongoing benefit from dinner meeting.	Not mentioned	Not mentioned	Not mentioned
M. Reilly-Smorawski et al. (2010) [56]	Two senior NICU staff nurses with backgrounds in psychology and social work	A closed, hospital-based format for couple-based support group(12 weeks): week 1–3: introductory phase week 3–11: open-format designweek 11: a qualitative evaluation tool was distributed and collected. week 12: summarizing the support group experience and for final preparation for life after the bereavement group. Leaders planned to offer to reconvene the group at intervals of 3 months for the year following the baby’s death	12 weeks couple-based bereavement group; attending weeklyTopics for discussion: A the baby’s death and related events B personal grief experiences C couple issues including gender-related grieving and communication D the future	Not applicable	Program was based on several observations on bereaved couples. Couple-based bereavement group was part of bereavement care program.	- After each 12-week session themes of the survey were bundled, and adjustments were made where needed to improve the support group functioning. - Education of the facilitators - Attending of bereavement counselling workshops and related conferences	Not mentioned
N. Snaman et al. (2017) [57], USA	Quality of life team, bereavement program coordinator and bereaved parent mentors	The bereavement program describes three parts: *Part 1:* Clinical and Supportive Interventions: - Child/family meet the QOL team and bereavement coordinator to start supportive relationship. Families receive a booklet, option for peer support. - Memorial event; two day gathering for bereaved parents whose child died 6 months to three years previously. *Part 2:* Parent-Created Materials: - Condolence card, several weeks to child’s death. - Bereavement resources guide is mailed within two weeks of a child’s death. - Seasons booklet & Remembrance mailings - Additional resources: books for siblings, parents videos. *Part 3:* Bereaved parent could be involved in education for staff and participate in research.	- Sending cards - Peer contact - Memorial day - Booklets and information folders - Video’s for parents - Contact by cards/emails	Not applicable	The program is developed by parents and staff. Bereaved parents and multidisciplinary members of the hospital comprise the Quality of Life (QOL) steering council under the guidance of an expert bereavement coordinator.	Parent mentors receive training on a variety of topics.	Not mentioned
O. Stastny et al. (2016) [58], USA	Public health nurse	After public health nurse has received information of coroner’s investigator families are contacted by phone/ email to schedule a home visit(s). Friends and family may be invited. During the home visit(s) the main focus is to provide support, education, SIDSs referrals, resources and connect with other SIDS bereaved families	- Phone contact - Home visit (Educate, support, provide resources, connect with peers, referral)	Not applicable	Authors experience (PHN SIDS coordinator)	Not mentioned	Not mentioned

### The characteristics of bereavement care interventions

The bereavement care programmes were predominantly initiated by hospital staff (A-N). They took place in the field of neonatology (*n* = 5) (F,H,I,M,O), paediatrics (*n* = 9) (B,C,D,E,G,J,K,L,N), or both neonatology and paediatrics (*n* = 1) (A). Some interventions were aimed at children with a certain diagnosis: Sudden Infant Death Syndrome (SIDS) (n = 1) (O), and cancer (*n* = 4) (B,E,G,N). Three studies presented a bereavement care programme, while focussing on the impact on HCPs of losing a patient (A,G,J).

With regard to the timing, we found that eleven interventions started after the child’s death (A,B,C,D,E,G,I,K,L,M,O), one intervention started during the end-of-life phase (J), and three interventions covered both before, and after, death (F,H,N).

In most interventions, the person intervening was either a nurse, appointed as the primary carer and operating individually or as part of a team (A,C,E,H,I,K,M), or a physician (A,C,D,G,I). Other people intervening included clinical social workers (B,H,K), chaplains (A,L) or peer supporters - parents who have previously lost a child too - (A), photographers (J), trained counsellors (D), public health nurses (O), team members who had the most contact with parents or experienced the lightest workload (F) or, bereavement care team members not otherwise specified (N).

We identified five overarching components of interventions which encompass the variety of practices described in the interventions. These are: (i) the acknowledgement of parenthood and the child’s life; (ii) establishing keepsakes; (iii) follow-up contact; (iv) education and information, and; (v) remembrance activities.

(i) The acknowledgement of parenthood and the child’s life consisted of washing, holding, or dressing the child (H,I), giving parents privacy in the moments surrounding the death of the child, for instance in a family room (H), providing the child with a certificate of life (I), or a blessing ceremony (F,H).
(ii) Establishing keepsakes consisted of safeguarding a lock of hair (H,I), hand, foot, or face print (H,I), pictures (F,H,I,J), or items that belonged to the child, such as toys, a blanket (H), ornaments (H), a memory stone (I), clothes (I), a baby ring or bracelet (H,I), memory books (F), poems (A,H), or other belongings (F,H). The created items were often provided to the parents in the form of a comfort basket or memory box (B,H). Keepsakes, especially for siblings, could also be provided (I).
(iii) Follow-up contact consisted of follow-up calls (A,B,E,F,G,H,I,K,O), cards (B,E,G,H,I,N), visits (A,F,L,O), flowers (F), condolence letters (K), and appointments (A,C,D,G,M). Follow-up contact also included facilitating contact with peers (A,K,N).
(iv) Education and information on coping, grief, and practical information concerning the death of the child, consists of folders and booklets with information (A,B,E,F,G,H,I,K,L,N), financial advice (F), videos containing information (L), educational support meetings for peers and relatives (L), seminars or workshops on coping and grief (K), and information sessions (A,C,D,G,M) during which HCPs provided information about the treatment and autopsy (I), or answered questions (I).
(v) Remembrance activities included ceremonies or services (F,H,K,N), and HCPs attending the funeral (E,L).

### The empirical basis of the interventions and the outcomes of the studies

Most interventions identified consisted of a description of practices, sometimes based on years of experience, but did not include an empirical or theoretical basis. Several studies did provide substantiation for their interventions such as a previous, non-specified, literature search (A,E), interviews and focus groups (B,E,G), or expert knowledge and special education (A,B,D,F,J,O). Only two interventions were developed using a clear theoretical basis. One intervention was based on principles of stress and social support theory (B), and the other contained a psycho-educational bereavement guide based on the principles of cognitive behavioural theory (K).

The studies that evaluated an intervention, showed that parents reported a positive experience with bereavement photography and follow-up contact (A,B,C,E,G,J,L). Parents were grateful to receive photos of their child, and helped HCPs feel better about their role (J). The outcomes of most of the empirical studies focused on how the parents had experienced the follow-up contact with the HCPs who had taken care of their child. Follow-up contact was generally valued. It helped parents cope with their grief, provided closure, and gave parents a secure feeling of the ongoing bond with the hospital and their child (A,B,C,E,G,L). Parents found follow-up meetings with HCPs and/or peers helpful in learning to tolerate and understand grief better. Moreover, it stimulated further thinking and discussion between the parents about the topics addressed in the meeting and helped parents to express their ideas and feelings concerning grief to each other and to their family and friends (L,M).

### The alignment between intervention components and theoretical key concepts

Given the lack of knowledge concerning the effectiveness of the interventions, the potential worth of the components of intervention is evaluated by aligning the five intervention components identified (i-v) to the key theoretical concepts as described in the Methods section. These are: anticipatory grief; attachment to working models and plans; appraisal processes; coping, and; continuing bonds. Hereafter, all the components will be discussed and hypothesised, considering how they align with the theoretical concepts identified (Table 4).

**Table 4:** The alignment of theoretical key concepts and intervention components

	Components concerning anticipatory grief	Components concerning attachment working models and plans	Components concerning the appraisal processes	Components concerning coping	Components concerning continuing bonds
Acknowledging parenthood and the child’s life	+	+		+	
Keepsakes		+		+	+
Follow-up contact		+	+	+	+
Education and information		+	+	+	
Remembrance activities				+	+

+: Intervention component supported by key theoretical concept

### The acknowledgement of parenthood and the child’s life

This component includes facilitating parents to fulfil their role as a parent, and to acknowledge the identity of their child. Facilitating parents in their parental role is a component HCPs provide before and after death. The main strategy in these interventions is to enable parents to nurture their child and to acknowledge their child’s uniqueness [54]. Parents are facilitated to experience the bond with the child, create memories, have a blessing ceremony, and say their farewells [59, 60]. It allows parents to begin to contemplate the idea that their child is dying, while ensuring that their child is as comfortable as possible [60]. These practices support anticipatory grief, since they foster emotional preparedness, allow parents to adjust slowly to the fact that their child is dying, and help to create lasting memories for parents to cherish after death [54]. A certificate of life empowers parents to recognise the identity of their child. In letting parents participate in the last care for their child, this also enables them to adjust, gradually, to the fact that their child is dying, and makes the transition between the internal plans less abrupt.

### Establishing keepsakes

HCPs take the initiative in creating keepsakes together with, or in accordance with, the parents. These keepsakes provide the parents with a tangible memory of the child. Especially in neonatology, where parents will not have been outside the hospital with their child, keepsakes provide parents with a way to cherish a part of their child, when the child is no longer present. Establishing keepsakes can help parents feel attached and close to their child and to provide comfort [54]. Over time, the keepsakes can help the parents in remembering the child, and help parents with processing, conceptually, the loss, while they revise the autobiographical memories and the memories of the child in order to adjust to the new reality. Over time, when the parents have adjusted to the new reality, the tangible memories of the child serve as a form for expressing the continuation of the bond between the parents and their child.

### Follow-up contact

Follow-up contact with the hospital may take various forms. Parents value ongoing contact with the hospital staff, since the hospital staff know the child and many parents developed a bond with them over time [45–47]. When parents feel that the HCPs remember their child, this is felt as an acknowledgement of the child’s identity, and a validation that their child has made an impact and mattered [45, 46]. This acknowledgement results in positive reappraisal processes and adds positive meaning to the past events. These positive reappraisals could also foster adaptive coping behaviours, for example the sharing of the story of the loss with friends and family. The continuous reappraisal and coping behaviours in turn result in altering the working models and plans because the loss is processed conceptually. This helps parents to find a place for, and to define a new bond with, the deceased child in the new reality [47]. Follow-up contact with HCPs and peer supporters, simply their presence and conversations, help parents to cope with loss [40, 43]. During follow-up contacts, HCPs can offer parents an explanation of the course of treatment and the rationale for certain decisions that were made. This is important as parents often describe being in a haze during the end-of-life period of their child [44, 46]. Furthermore, autopsy results are often shared in order to clarify the physical illness [53, 54]. HCPs also have the opportunity to reassure parents that there is nothing that they could have done differently [58]. This helps parents to make sense of the preceding events and to clarify the memories surrounding the death of their child [46, 53]. This clarification, in turn, aids reappraisal of the situation and past events, and provides parents with a form of closure. It also allows parents to readjust their memories of the situation, address doubts about themselves, and treasure memories of their child, which results in readjustment to new memories and thus creates new plans about themselves, their child, and the past events.

### Education and information

Information folders, booklets, workshops, and seminars can help parents in regaining some control over the many different challenges they face in a new, unknown, and insecure, situation. It makes parents feel more prepared in practical terms such as with financial aid, funeral arrangements, and in finding extra emotional assistance when needed [59]. An example of practical assistance might be how to provide explanations to, and support for, the siblings, reassuring parents that what they are feeling is normal, actions which can be termed preparation and which offer a sense of validation [55, 59]. But practical assistance could also include providing information about when and who to turn to for extra support [55]. These forms of assistance support parents in coping with the new situation because it makes the new demands slightly more manageable. The information provided, and the validation of the emotions they experience, also assist parents in creating new knowledge structures and plans with regard to their grief and the future they face. It helps the appraisal processes and offers new working models.

### Remembrance activities

The remembrance activities provide an opportunity to feel close to the child again and to recollect memories about their life [60]. It is also a means of feeling supported by friends, family, hospital staff, and the community, that may help parents to cope with the loss [51]. These remembrance occasions provide a secure environment where parents feel connected to the child and feel the bond that they had, and that still exists. Remembrance activities help parents in finding a way to continue their bond with the child in the new reality. Religious or spiritual aspects of the events can also help parents to make sense of, and find meaning in, the child’s death. Such “meaning making” after the death is a helpful coping mechanism for parents, in which they can revise their memories and plans surrounding the death of their child in a positive and helpful manner.

## Discussion

This review identified fifteen well-defined bereavement interventions provided by regular HCPs to support parents of seriously ill children both at the end of their child’s life and after death. All interventions were clustered into five overarching components of the intervention. These are: the acknowledgement of parenthood and the child’s life; establishing keepsakes; follow-up contact; education and information, and; remembrance activities. The majority of interventions started after the death of the child, and were performed by a nurse, assigned as the primary carer, or a physician. Most of the empirical studies included in this review evaluated how to conduct the intervention and experiences with the interventions, but not their effectiveness. To compensate for this lack of evidence, the components of intervention were assessed against a theoretical synthesis on loss and grief, which revealed that all the components from which the interventions were built were covered by theories on a conceptual level. The theoretical synthesis did uncover that bereavement is characterised by the continuous process of adjusting to a new reality [18–21, 23, 26–30]. Five key theoretical concepts clarify this process: anticipatory grief; attachment working models and plans; the appraisal processes; coping behaviours, and; continuing bonds. The theoretical synthesis shows the need for bereavement interventions to focus on the continuous nature of grief, and thus, starting before the death and guiding parents through the grieving process. Most interventions we identified relied on a combination of multiple components or time points. However, few interventions reviewed here showed such a continuous process in supporting the parents.

In our comparison of the components of intervention, and the theoretical synthesis, we found HCPs pursued several underlying aims for providing bereavement care to parents. The interventions were offered by HCPs to enhance the parents’ feeling of preparedness towards the death of their child. These comprise providing parents with information, nurturing the child, and experiencing support from HCPs or their peer supporters. Those designed to enhance their ability to create memories of, and with, their child include nurturing the child, treasuring keepsakes, and recollecting memories at the subsequent remembrance ceremony. Finally, the interventions to provide parents with comfort and reassurance involve making memories and keepsakes, answering questions and providing comfort in follow-up, providing information in general, and remembering and acknowledging the child. These elements are not captured in a single moment, but require support at different moments and in a continuous nature [61]. A difference we noticed is that the importance of supporting parents in their parental role, and acknowledging the identity of the child, may have a different meaning in neonatology compared to paediatrics [54, 62]. The time in the hospital is often the only time these parents can make memories with their child and to nurture them. The HCPs are often the only people, apart from the family, to have seen the child alive.

Bereavement theories emphasise that dealing with loss takes form in a transition towards a new reality [18–21, 23, 26–30]. However, only four interventions included in this review commenced before the death of the child [50, 57, 59, 60]. Yet, conversations between HCPs and parents about the condition of their child, and their preparedness for the death of their child, can contribute positively to the bereavement process after their child has died [25, 63]. The possible explanations for this are, firstly, that there is a delicate balance between preserving hope and letting go of the child during the end-of-life phase. Most, but not all, parents are able to make this transition [4, 64]. Most parents are intellectually aware that their child’s death is imminent, however, emotional awareness usually follows at a later stage, or not until after the death [65]. For the HCPs these phenomena, and the parental diversity, make it difficult to assess when parents are receptive to bereavement support during the end-of-life phase. Furthermore, this diversity tends to provoke insecurity among HCPs. However, HCPs should be able to influence parents’ awareness and openness towards bereavement support, for example by informing parents about the finality of curative options by sharing information honestly and considering whether to stop ongoing curative treatment [65]. Secondly, given the diversity both in parental responses to letting go of their child, and in their emotional awareness, it is difficult to create a standardised intervention, including a protocol, for bereavement care for parents during the end-of-life phase. Since our inclusion criteria consisted of interventions that needed to be replicable, and supported by a protocol or documents, these kind of interventions could have been excluded. This could mean that there is, in fact, attention for feelings of loss and grief, prior to the death of the child, by HCPs in their current daily practice. However, these practices are not standardised and thus were not covered in this review.

The comparison of key theoretical concepts and components of intervention showed that interventions all account for small fragmented pieces in the grieving process. But, also, that there are no interventions that emphasise the continuous parental adjustment process as a whole. The regular HCPs who had been involved in the child’s care since diagnosis could be a significant factor in this continuous care. Studies have shown that parents require at least one meaningful follow-up contact with the HCPs who cared for their child [14, 66]. We propose that bereavement care, including follow-up conversations, are important parts of the regular HCPs’ activities. There are three main reasons for the integration of follow-up care into the HCPs activities. Firstly, parents often have outstanding questions about their child’s care, illness, and their role in the period of the illness [67]. The regular HCPs are able to answer these questions since they have been part of the care prior to death. Secondly, the trustworthiness and bonds that already exist between the HCPs and parents are very important [54]. Thirdly, parents seek proximity to their child - an acknowledgement of his or her life, and the impact the life has made; it helps parents in the grieving process when the HCPs speak of their memories of the child, reflect on his or her unique identity, and are effected by the child’s death [14, 45]. Another important element of the conversations between the HCPs and parents could be psycho-education [68, 69]. Psycho-education encompasses information about what parents are experiencing while preparing them for what they could encounter during their journey through the grieving process. It has been shown to have positive effects on the self-efficacy of informal caregivers. Psycho-education could strengthen parents in their transition to a new reality where the child is no longer physically present, if they understand which challenges they are going to face, and prepare them with helpful coping strategies [68]. Psycho-education might too have a positive effect on mental appraisals when a setback in the grieving process occurs and in validating the feelings parents experience as normal [70].

Once a child dies, their parents are left with an overwhelming sense of grief. They describe the time passing as a blur [44, 54]. Parents are not aware, during that period, of all the interventions and assistance HCPs could offer them. However, options could be presented to parents, and the most appropriate could be chosen. Therefore, it is important that HCPs offer parents a broad range of interventions [71]. This is also important because the key theoretical concepts are not sequential. Instead they form a continuum and the most dominant of these key concepts alter according to the demands at a given time [18, 20, 21, 27]. Also, effective coping is defined by a process of alternating between two or more different coping strategies, depending on the demands at a specific time [72]. If HCPs could determine, in what stage parents were at a given time, or with which processes they experience difficulties, the appropriate components of intervention to aid that process could be selected.

### Strengths and limitations

The search was constructed using a recently developed method, PALETTE, in addition to PRISMA. This was helpful in identifying all the relevant articles in relatively young domains where terminology is still diffuse. To our knowledge, given the difficulty of measuring outcomes in the field of paediatric palliative care, this is the first systematic review to give insight into the theoretical effectiveness of bereavement interventions. In particular, the inclusion of replicable interventions provides HCPs with opportunities to implement them in their practice. A limitation of this systematic review concerns the inclusion and exclusion criteria. These eliminated less developed practices and potentially helpful professional attitudes and behaviours out of sight. It is possible that these contain strategies that can be considered supportive in parental grief. Also, we included replicable interventions which could be implemented in practice since these interventions are supported by a protocol or clear guidelines. However, most interventions are not tested and offer little evidence in their support. This is required before implementing an intervention. Testing these interventions might then be difficult due to the setting of paediatric palliative care. Therefore, the theoretical synthesis and alignment could only provide a form of theoretical support for the interventions we reviewed.

## Conclusion

This review provides an overview of well-defined, replicable, bereavement interventions. The theoretical synthesis in this review provides a basis for the effectiveness of the components of intervention. All five of these cover multiple key concepts derived from theory. HCPs can choose multiple interventions for different components to provide parents with a continuous form of bereavement care, aiding the transition that parents have to go through following their loss. Future research is needed on how this continuous support can be established, which time points are crucial for providing bereavement care, and how new interventions can be developed that align with this transition, and thus, ultimately, help parents in adjusting to their new reality.

## Supplementary information

**Additional file 1.** Search strategy.

**Additional file 2.** Synthesis of theories on grief and loss.

## Data Availability

Not applicable.

## Abbreviations

COREQ: COnsolidated criteria for REporting Qualitative research
HCP(s): Health Care Professional(s)
NICU: Neonatal Intensive Care Unit
PALETTE: Palliative cAre Literature rEview iTeraTive
PICU: Paediatric Intensive Care Unit
PRISMA: Preferred Reporting Items for Systematic Reviews and Meta-Analyses
SIDS: Sudden Infant Death Syndrome
