# Supplementary material for: When a child dies: a systematic review of well-defined parent-focused bereavement interventions and their alignment with grief- and loss theories
Source: BMC Palliat Care. 2020 Mar 12;19:28. doi: 10.1186/s12904-020-0529-z (PMC7068872; doi:10.1186/s12904-020-0529-z)
Supplement: Supplementary file 1 — Additional file 1. Search strategy. [file 12904_2020_529_MOESM1_ESM.docx]

**Additional file 1.** Search strategy.

| Search strategies for all databases, search performed on November 15, 2018  Ovid MEDLINE(R) ALL <1946 to November 15, 2018>  (((exp *health personnel/ or (((health or healthcare) adj3 (assistant or provider or professional* or personnel or staff)) or caregiver* or care-giver* or nurse* or nursing or physician* or clinicians* or surgeon* or pediatric* or paediatric* or neonatologist* or NICU).ti,ab,kf.) and (exp *parents/ or (mother* or father* or family or families or couple* or parent*).ti,ab,kf.)) or exp *Professional-Family Relations/) and (exp *bereavement/ or (bereave* or grief or grieving or mourning or mournful or mourned or "death of a loved one" or "premature demise" or ((child or patient) adj3 death)).ti,ab,kf.)  Embase Classic+Embase <1947 to 2018 November 15>  (((exp *health care personnel/ or (((health or healthcare) adj3 (assistant or provider or professional* or personnel or staff)) or caregiver* or care-giver* or nurse* or nursing or physician* or clinicians* or surgeon* or p?ediatric* or neonatologist* or NICU).ti,ab,kw.) and (exp *parent/ or (mother* or father* or family or families or couple* or parent*).ti,ab,kw.)) or *human relation/) and (exp *bereavement/ or exp *grief/ or (bereave* or grief or grieving or mourning or mournful or mourned or "death of a loved one" or "premature demise" or ((child or patient) adj3 death)).ti,ab,kw.)  CINAHL seach: limitations - MEDLINE searchmode - Booleaans/frase  ((((MM "Health Personnel+") OR AB(((health or healthcare) N3 (assistant or provider or professional* or personnel or staff)) or caregiver* or care-giver* or nurse* or nursing or physician* or clinicians* or surgeon* or pediatric* or paediatric* or neonatologist* or NICU) or TI(((health or healthcare) N3 (assistant or provider or professional* or personnel or staff)) or caregiver* or care-giver* or nurse* or nursing or physician* or clinicians* or surgeon* or pediatric* or paediatric* or neonatolog ...) AND ((MM "Parents+") or AB(mother* or father* or family or families or couple* or parent*) or TI(mother* or father* or family or families or couple* or parent*))) OR (MM "Professional-Family Relations"))  AND  (MM "Bereavement+") OR (MH "Grief+") or AB(bereave* or grief or grieving or mourning or mournful or mourned or "death of a loved one" or "premature demise" or ((child or patient) N3 death)) or TI(bereave* or grief or grieving or mourning or mournful or mourned or "death of a loved one" or "premature demise" or ((child or patient) N3 death)) |
| --- |
