# Supplementary material for: When a child dies: a systematic review of well-defined parent-focused bereavement interventions and their alignment with grief- and loss theories
Source: BMC Palliat Care. 2020 Mar 12;19:28. doi: 10.1186/s12904-020-0529-z (PMC7068872; doi:10.1186/s12904-020-0529-z)
Supplement: Supplementary file 2 — Additional file 2. Synthesis of theories on grief and loss. [file 12904_2020_529_MOESM2_ESM.docx]

**Additional file 2.** Synthesis of theories on grief and loss.

|  | *Components concerning anticipatory grief* | *Components concerning attachment working models and schemas* | *Components concerning appraisal processes* | *Components concerning coping* | *Components concerning continuing bonds* |
| --- | --- | --- | --- | --- | --- |
| Boelen^18^ |  | A need to integrate the reality of the loss into the person’s existing mental representations of the self, the world, and the relationship with lost person. | Maladaptive beliefs (about the self, life, the future, the world, and one’s own responses to the loss) need to be changed into beliefs that foster adjustment. | Anxious and depressive avoidance strategies need to be replaced by more helpful strategies that facilitate adjustment. |  |
| Bowlby^19,23^ |  | A disruption of attachment working model results in a need for reorganizing representations of the lost person and the self. | Awareness of a constant mismatch between existing schemas and reality. |  |  |
| Folkman^24^ |  |  | Ongoing cognitive reappraisals of the stressful situation. | Dealing with the stressful situation using problem- and emotion-focused coping strategies. |  |
| Hebert^25^ | Preparedness as an important factor in end-of-life care. Importance of preparedness in the medical, psychosocial, spiritual, and practical dimensions |  |  |  |  |
| Horowitz^26^ |  | Need for reschematization of the person- and relation schemas after the loss. | In the process of reschematization, awareness of the inability of previously held knowledge structures to account for new information, and time to develop new meaning structures is needed. | The reaction of the person depends on how the event and context interact with preexisting personality structures and coping styles. | Reschematization accounts for the relationship with the deceased, and the attachment bond to live on in the mind. |
| Maccallum^27^ |  | Revision of self-identity is needed to incorporate the reality of loss in the autobiographical memory. | Adjustment to loss requires that roles, personal goals, motivations, and future plans that were shared with the deceased are no longer entirely based on the deceased | Emotion regulation strategies are influenced by the self-identity. Need for flexible use of emotion regulation strategies. |  |
| Neimeyer^28,29^ |  | Importance of meaning making processes of the death. Need for change in (inter)personal systems of meanings and find significance both in the death and in own ongoing life. |  | Meaning-making is a process and differs over time, from finding an answer to the “why” of the death to the positive benefits of the loss for survivors. |  |
| Rando^30^ | Anticipatory mourning is experiencing loss or trauma in the awareness of life-threatening or terminal illness. Consists of: grief and mourning, coping, interaction, psychosocial reorganization, planning, balancing conflicting demands, and facilitating an appropriate death. | Involves readjustment to the new world without forgetting the old world, developing a new relationship with the deceased, and forming a new identity. | Revision of the assumptive world, and adopting new ways to be in the new world. | During anticipatory mourning, coping can be directed toward disease related, dying related, or ongoing life processes. |  |
| Shear ^20^ |  | Stress response because of absence of attachment figure, resolves when the permanence of the loss is integrated into the long-term memory and attachment representations are updated. | Continuous mismatch between the mental representation of the attachment figure and the reality of their death, this causes stress. | Stress activates the attachment system. However, unavailability of the attachment figure causes (even) more disruption of the effective functioning of the emotion regulation system. There is a need for effective coping strategies. |  |
| Stroebe^21,22^ |  |  |  | Adaptive coping with grief by alternating between confrontation and avoidance strategies, depending on the situation. | During loss orientation, a rebuilding of assumptions about the presence of the lost person in one’s life takes place. |
